# Development and Optimisation of Hydrogel Scaffolds for Microvascular Network Formation

**DOI:** 10.3390/bioengineering10080964

**Published:** 2023-08-15

**Authors:** Carla V. Fuenteslópez, Mark S. Thompson, Hua Ye

**Affiliations:** Institute of Biomedical Engineering, University of Oxford, Oxford OX3 7DQ, UK; carla.fuenteslopez@eng.ox.ac.uk (C.V.F.); mark.thompson@eng.ox.ac.uk (M.S.T.)

**Keywords:** microvascular network, hydrogel, tissue engineering scaffold, traumatic injury, fibrin, collagen, gelatine

## Abstract

**Highlights:**

- This research presents the development of a hydrogel scaffold for an in vitro microvascultature. Here, the optimal hydrogel composition in terms of polymer type, ratio, and solvent is investigated.
- Scaffold optimisation for endothelial cells is based on a multipronged evaluation encompassing seeding number, cell adhesion, migration rate, cell viability, hydrogel consistency, and endothelial tube formation.
- The developed hydrogel constructs enabled the formation of interconnected capillary-like networks, which are also characterised to gain further insights into their microarchitecture.

**Abstract:**

Traumatic injuries are a major cause of morbidity and mortality worldwide; however, there is limited research on microvascular traumatic injuries. To address this gap, this research aims to develop and optimise an in vitro construct for traumatic injury research at the microvascular level. Tissue engineering constructs were created using a range of polymers (collagen, fibrin, and gelatine), solvents (PBS, serum-free endothelial media, and MES/NaCl buffer), and concentrations (1–5% *w*/*v*). Constructs created from these hydrogels and HUVECs were evaluated to identify the optimal composition in terms of cell proliferation, adhesion, migration rate, viability, hydrogel consistency and shape retention, and tube formation. Gelatine hydrogels were associated with a lower cell adhesion, whereas fibrin and collagen ones displayed similar or better results than the control, and collagen hydrogels exhibited poor shape retention; fibrin scaffolds, particularly at high concentrations, displayed good hydrogel consistency. Based on the multipronged evaluation, fibrin hydrogels in serum-free media at 3 and 5% *w*/*v* were selected for further experimental work and enabled the formation of interconnected capillary-like networks. The networks formed in both hydrogels displayed a similar architecture in terms of the number of segments (10.3 ± 3.21 vs. 9.6 ± 3.51) and diameter (8.6446 ± 3.0792 μm vs. 7.8599 ± 2.3794 μm).

## 1. Introduction

Microvascular traumatic injuries are common but relatively understudied [1]; whereas the combined impact of traumatic injuries, both in terms of mortality and morbidity, is largely unknown, an estimate is calculated using the disability-adjusted life year methodology that considers both the years of life lost and years lived with disability [2]. The most recent Global Burden of Disease report estimates that injuries account for 7.6% of all deaths and 11.0% of Years of Life Lost [3].

Major trauma has often been perceived to be a ‘disease of the young’ [4]. However, the prevalence of traumatic injuries has increased in the elderly [5], partly due to the ageing population and an increased exposure to the risk of injury and frailty due to age [6,7].

In addition to clinical research, the vast majority of experimental methods used to study traumatic injuries rely on in vivo models [8]. Nevertheless, these entail major ethical concerns and display physiologic and pathophysiologic differences [8], which complicate translational research in trauma. Moreover, utilising current models in traumatic injury research is associated with a number of challenges, including enabling microvasculature formation within a 3D structure.

As such, there is an urgent need to develop physiologically relevant in vitro models that allow us to gain fundamental understanding on trauma and, at a later state, test potential therapeutics to enhance tissue repair and regeneration. To address this gap, this research aims to develop and optimise a construct for microvascular network formation.

In an effort to mimic the natural extracellular matrix (ECM), biological substitutes are specially designed to provide a platform that offers specific characteristics for a particular application [9]. These scaffolds play a significant role in providing support, achieving a particular architecture, and allowing for certain physical and biological functions when interacting with cells. The 3D matrices with structural, mechanical, and chemical properties that resemble the natural environment have allowed for the maturation of functional tissue engineering, where biological constructs mimic load-bearing tissues [10].

Engineering perfusion in in vitro models is one of the major challenges of tissue engineering [11]. Most in vitro constructs do not contain microvascular structures as occurs in the native tissue, so cells in those scaffolds rely to a large extent on simple diffusion for oxygenation and nutrient delivery [11,12]. The lack of a vascular network is partially responsible for the limited size of in vitro engineered tissues, such as skeletal muscle [13].

Broadly, the microvascular in vitro models that have been developed to date can be grouped into four general categories: microfluidics, templating, 3D printing, and self-organisation. These have a tendency towards either organogenesis or organ-on-a-chip technology and, as such, are associated with different degrees of complexity, throughput, and physiological relevance.

Self-organised microvascular models, where guided capillary self-organisation and angiogenesis can take place [14], have been developed using human umbilical vein endothelial cells (HUVECs) [15]. Relevantly, these have been used to form microvessels with a diameter ranging between 10 and 100 μm. Furthermore, self-organised microvascular models allow for interconnected networks [16]. The majority of these in vitro models have been used in pharmaceutical [17] or cancer research [18].

The design of microvascular in vitro models encompasses not only the architecture of the scaffold but also the selection and optimisation of materials. ECM-mimetic materials have been used to fabricate hydrogels [19]. Polymers in hydrogel format not only partially resemble the physical characteristics of native ECM [20,21,22], but they can also be fine tuned to achieve the desired properties by adjusting its parameters, such as concentration [20,23].

A range of natural and synthetic polymers have been used to fabricate hydrogels [23], including fibrin [19,24,25], hyaluronic acid [19,26], chitosan [27], collagen [28,29], alginate [30,31], and gelatine [32,33]. Hydrogels created from naturally derived materials have inherent biocompatibility, bioactivity, and biodegradability [20], thus offering promising alternatives to fabricate tissue engineering scaffolds.

Also, 3D scaffolds have been crafted using Matrigel to provide structural support, as it closely mimics the mechanical and chemical properties of the native ECM [34]. However, its components are poorly defined [34], it is associated with high variability between different batches [35], and its mechanical properties are heterogeneous throughout the samples [36]. In contrast, natural hydrogels such as collagen are associated with significantly higher consistency and reproducibility while maintaining a good biological relevance [35].

The research presented here further investigates three natural polymers—collagen, fibrin, and gelatine—to fabricate hydrogel constructs. Collagen is one of the most abundant fibrous proteins in the body, providing biological and mechanical support, and one of the major elements of the ECM [20,37]. Fibrin is also a major protein in ECM and plays a role in a number of bioactivities including angiogenesis [25]. Gelatine facilitates cell adhesion as it contains specific peptide sequences allowing for integrin cell receptor recognition [33]. Relevantly, these polymers have demonstrated to have excellent biocompatibility and have been used to fabricate hydrogels mimicking the native ECM [20,33].

## 2. Materials and Methods

### 2.1. Cell Culture

Human umbilical vein endothelial cells (HUVECs; ATCC) were cultured in T-25 and T-75 cell culture flasks (Corning Incorporated-Life Sciences, Oneonta, NY, USA) with Endothelial Cell Growth Medium-2 (EGM-2) supplemented with SingleQuots (Lonza, Walkersville, MD, USA), and 1% penicillin/streptomycin (Gibco Life Technologies, Grand Island, NY, USA). Cells were incubated at 37 °C and 5% $CO_{2}$. Medium was changed every 48–72 h, as required. Cells were used for experimental work at 80% confluency and up to passage number 7.

### 2.2. Fabrication of Hydrogels

A range of hydrogels was prepared at 1%, 1.5%, 2%, 2.5%, 3%, or 5% weight/volume (*w*/*v*) in phosphate-buffered saline (PBS; Gibco Life Technologies, Paisley, UK), serum-free EGM-2 media, or an MES/NaCl buffer (for fibrin only). Following gel casting, plates were incubated at 37 °C for 30 min. Then, they were sterilised under UV light for 20 min before cell seeding.

#### 2.2.1. Collagen

An adaptation of a protocol described by Lim et al. [38] was used to prepare the collagen gels. Collagen type I from rat tail (Sigma Life Sciences, St. Louis, MO, USA) was mixed with serum-free media or PBS to obtain the desired concentration. pH was measured with a pH meter and adjusted to pH 7.4 using NaOH drops. After casting, collagen hydrogels were cross-linked by irradiating the samples with UV light for 20 min.

#### 2.2.2. Gelatine

Gelatine from porcine skin (Sigma Life Sciences, St. Louis, MO, USA) was dissolved in warm serum-free media or PBS, magnetically stirred until fully dissolved, and filtered twice (20 μm filter). Hydrogels were cross-linked by UV irradiation (90 min).

#### 2.2.3. Fibrin

Fibrin gels were prepared based on a protocol from Lim et al. [38] and Sharma et al. [39]. Briefly, a fibrinogen (MP Biomedicals, Auckland, New Zealand) solution at the desired concentration was prepared in MES/NaCl buffer (0.15 M NaCl, 2.5 Mm MES in water), PBS or serum-free media and stored at 37 °C for 30 min or until fully dissolved. pH was adjusted to 7.4 using NaOH drops. To prepare the gel, the fibrin solution was cross-linked with thrombin (Sigma-Aldrich, St. Louis, MO, USA) at a 20:1 volume ratio, and a 1 M CaCl${}_{2}$ solution in water was added to the mixture at a volume ratio of 1:100 to thrombin. The solution was pipetted to ensure a homogeneous mixture and quickly poured into the wells as the gelation process begins when the three ingredients come together.

### 2.3. Cell Proliferation

HUVECs (P6) were seeded at a density of 0.5, 1, 1.5, or 2 × $10^{4}$ cells per well (96-well plate) and cultured with supplemented EGM-2 media for 48 h at 37 °C and 5% CO${}_{2}$. A total of 22 replicates of each experimental condition and control were used. Media was replaced every 24 h, and cell proliferation was counted at 24 h and 48 h after seeding. Cells were counted using a MicroPlate reader (SpectraMax i3x, Molecular Devices, LLC., San Jose, CA, USA) and its software (SoftMax Pro; Version 7.0, Molecular Devices, LLC., San Jose, CA, USA). Cell counts were validated by manually counting five randomly selected wells.

### 2.4. Cell Adhesion

Wells were coated with gelatine, fibrin, or collagen in PBS, serum-free media, or MES/NaCl (fibrin only) at a concentration of 1, 1.5, 2, 2.5, 3, or 5% *w*/*v*. The hydrogel suspension was carefully pipetted into the well and plates were incubated at 37 °C for 30 min. Following excess removal, a thin coat covering the bottom of the well remained. Plates were then sterilised (UV light, 20 min) and washed thrice with PBS. HUVECs (P6) were seeded at a density of 2 × $10^{4}$ cells/well (96-well plate) and cultured with supplemented EGM-2 media at 37 °C and 5% CO${}_{2}$.

At 4, 6, 8, and 24 h, the supernatant was discarded, and wells were refilled with fresh supplemented EGM-2 media. Cells were counted using a MicroPlate reader and randomly chosen wells were validated against manual counting from the images obtained. Four replicates of each coating and of the control with no coating (i.e., cells only) were used.

### 2.5. Wound Healing

A wound-healing assay, also known as a scratch assay, was conducted using HUVECs plated on coated wells to compare cell migration rates across hydrogels based on the protocol by Jonkman et al. [40]. Wells were prepared similarly to those described in the adhesion experiments. HUVECs (P4) were seeded at a density of 1.5 × $10^{4}$ cells/well and after reaching confluency, a p20 pipette tip was used to scratch a ‘wound’ (average initial gap area: 2.2098 mm${}^{2}$, standard deviation 0.4201 mm${}^{2}$) across the monolayer. Then, cell migration was monitored at 0.5, 1, 1.5, 2, 3, 6, 8, 12, and 22 h. Triplicates of each experimental and control variations were used.

The gap area was measured using ImageJ and with these data, cell migration rate ($v_{migration}$; Equation (1)) and time taken to fill the gap (Equation (2)) were calculated. The gap area was plotted for each time point, and a straight-line fit was used to obtain the trendline. From here, the slope was used to calculate the wound-healing rate and, in conjunction with the initial gap area, the time it takes for the gap to be filled.

(1) $$v_{migration}=\frac{\left|slope\right|}{2\times length\;of\;gap}$$

(2) $$t=\frac{Initial\;Gap\;Area}{2\times |slope|}$$

### 2.6. Cell Viability

Fibrin and collagen gels were prepared as previously described and were then carefully pipetted into wells to avoid bubble formation. The collagen–fibrin hydrogels were prepared at a 1:1 ratio. Plates were incubated at 37 °C for 1 h to allow for gelation. Before cell seeding, plates were placed under UV light for 20 min for sterilisation purposes. HUVECs (P6) were seeded on top of the gels (seeding density: 2 × $10^{4}$ cells/well) and cultured with supplemented EGM-2 media for up to 96 h at 37 °C and 5% CO${}_{2}$. Triplicates were prepared, including cells-only controls.

Cell viability was measured every 24 h using a live/dead viability kit and a microplate reader. A live/dead viability kit (Molecular Probes Invitrogen, Eugene, OR, USA) was used following the manufacturer’s instructions. Briefly, 20 μL of a 2 Mm EthD-1 solution and 5 μL of a 5 Mm Calcein AM solution were added to 10 ml of PBS. This solution was added to the cells following media removal and incubated for 30 min at room temperature, away from the light. Calcein AM was used to stain live cells green, whereas dead cells were stained red using EthD-1. A microplate reader (SpectraMax i3x, Molecular Devices, San Jose, CA, USA) was used to measure the fluorescence of Calcein AM (530 nm, excited at 645 nm) and EthD-1 (485 nm, excited at 530 nm), separately, for each sample. Background fluorescence, both from the plate and wells with a layer of gel but with no cells, was subtracted from the readings prior to the calculation of the live cell percentage (Equation (3)).

(3) $$Live\;Cells\;\left(\%\right)=\frac{F{\left(530\right)}_{sample}-F{\left(530\right)}_{min}}{F{\left(530\right)}_{max}-F{\left(530\right)}_{min}}\times 100$$

### 2.7. Hydrogel Consistency

Collagen, fibrin, and collagen–fibrin in PBS, media, or MES/NaCl (fibrin only) hydrogels were cast in 96-well plates and incubated at 37 °C for 1 h. Then, each sample was carefully removed from the wells and placed on a Petri dish. After 1 h, the samples were visually inspected and each hydrogel composition was classified as poor, acceptable, or good based on shape retention once removed from the moulds.

### 2.8. Tube Formation

A tube formation assay was carried out to evaluate the impact of the base gel in capillary-like structure formations. Fibrin in serum-free media (3% and 5% *w*/*v*) hydrogels were prepared to evaluate tube formation.

Cells were grown using supplemented media and, once confluent, they were used for experimental work. Serum-free media was used from here on. Separately, fibrin was prepared as previously described and 75 μL was added to each well (96-well plate). Careful attention was paid to ensure that the hydrogel was evenly distributed and that no bubbles were formed. Plates were placed in a humidified incubator (37 °C, 5% CO${}_{2}$) for 1 h to allow for gelation. Before seeding the cells on top of the gel, plates were placed under UV light (20 min) for sterilisation.

**Figure 1:**
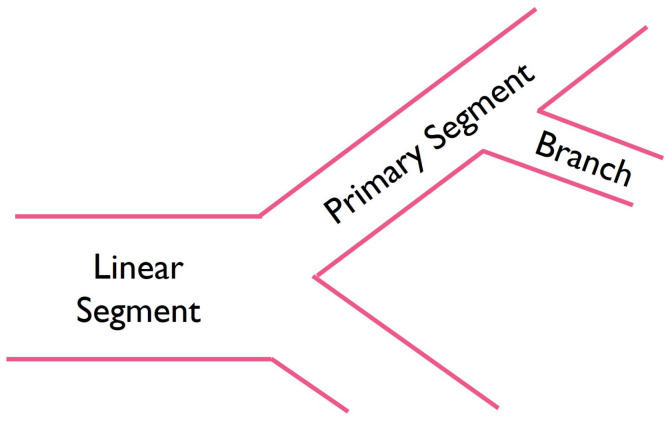
Schematic illustration of microvascular segments.

Cells were carefully pipetted on the hydrogels, at a density of 2 × $10^{4}$ cells/well, and were incubated. Tube formation was monitored every hour until a full network of capillary-like structures developed. Length and diameter of each segment were measured and classified (Figure 1) as a linear segment (i.e., when a single tube connects two points), a primary segment (i.e., segment from which other segments originate), or a branch (i.e., segments that arise from primary segments). The branch to primary segment ratio and the angles between these were also quantified. ImageJ/Fiji (version 1.54d, National Institutes of Health, Bethesda, MD, USA) [41] was used for image analysis.

### 2.9. Statistical Analysis

Where relevant, data were evaluated using one- or two-way analysis of variance (ANOVA) with post hoc tests using Tukey’s honest significant difference (HSD), where *p* values ≤ 0.05 were deemed to be statistically significant. Data analysis was performed using Excel 2022 (version 2208, Microsoft, Redmond, WA, USA). Unless otherwise specified, data are presented as mean values with standard deviation. Figures were created using GraphPad Prism (version 9, Dotmatics, Boston, MA, USA).

## 3. Results

### 3.1. Cell Proliferation by Seeding Density

**Figure 2:**
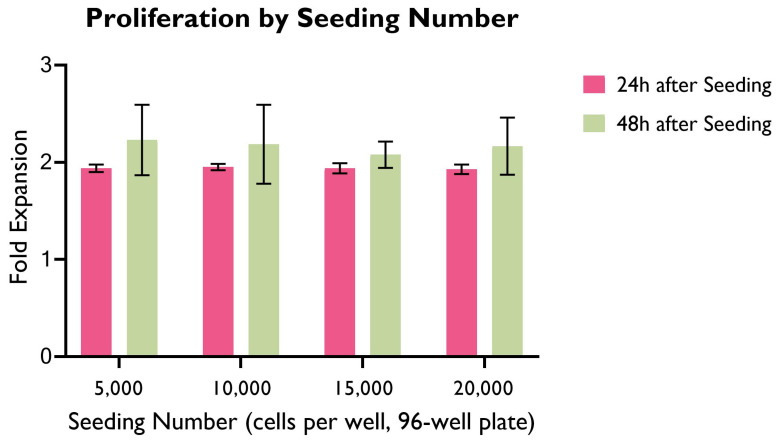
Cell proliferation as fold expansion at 24 h and 48 h, by seeding density. HUVECs were cultured in supplemented EGM-2 media in 96-well plates. (ANOVA, *p* > 0.05; *n* = 22).

Cell proliferation of HUVECs seeded at different seeding densities was measured at 24 h and 48 h (Figure 2), and was graphed as a fold expansion of the initial seeding number for each time point.

Across all seeding densities, the highest cell proliferation as a fold expansion occurs in the first 24 h after seeding, whereas the variation between 24 h and 48 h is significantly smaller. The highest variation is 40.91% (seeding density: 1.5 × $10^{4}$ cells/well) and the lowest is 33.37% (seeding density: 0.5 × $10^{4}$ cells/well).

Interestingly, there were no significant differences between groups in the first 24 h. This could potentially suggest that the cell proliferation rate does not vary greatly based on cell seeding density within this period. It is worth highlighting that in a longer period, cell proliferation as a fold expansion of the seeding number might reach a plateau due to the constraints imposed by the limited space and cell culture media available to cells.

Despite not producing statistically significant differences, 1.5 × $10^{4}$ or 2 × $10^{4}$ cells/well were preferred for further experimental work as these allowed for a higher cell count in a shorter period of time, therefore accelerating experimental work. Careful monitoring of cell proliferation, particularly when confluency is high, is required to avoid cell overcrowding and death.

### 3.2. Cell Adhesion

**Figure 3:**
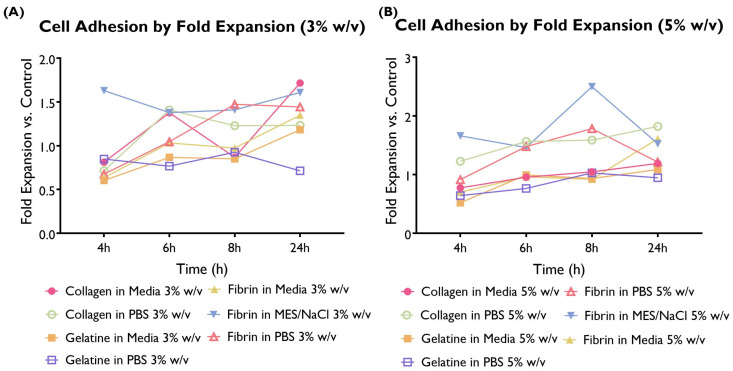
Cell adhesion as a fold expansion of the cells-only control by coating at a concentration of 3 or 5% *w*/*v*. HUVECS were cultured in supplemented EGM-2 and seeded at a density of 2 × $10^{4}$ cells/well (96-well plate). A total of 14 hydrogel compositions had statistically significant differences compared to the control. A total of 11 compositions exhibited higher cell adhesion (collagen in media 1, 1.5, and 2.5%; collagen in PBS 5%; fibrin in media 1%; fibrin in MES/NaCl 1.5, 2, 2.5, 3, and 5%; and fibrin in PBS 5%), whereas only three variations had significantly lower values (collagen in PBS 2%; fibrin in PBS 1%; and gelatine in PBS 1%). (ANOVA/Tukey HSD, *p* < 0.05; *n* = 4).

Cell adhesion to the different hydrogel compositions was compared with the cells-only control and illustrated here as a fold expansion of the cell adhesion exhibited by the control (Figure 3).

The differences in terms of cell adhesion between hydrogel compositions were statistically significant. When looking at the overall performance across time points, 11 hydrogel compositions were associated with a statistically significant higher cell adhesion than the control, whereas only three compositions were lower. The remaining hydrogel compositions were associated with similar cell adhesion to the control. Collagen in media (predominantly 1, 1.5, and 2.5%), fibrin in media (predominantly 1%), and fibrin in MES/NaCl (predominantly 1.5–5%) performed particularly well. On the other hand, gelatine in both serum-free media and PBS shows lower adhesion than the control. This suggests that gelatine is not particularly well suited for the development of this construct.

### 3.3. Wound Healing

**Figure 4:**
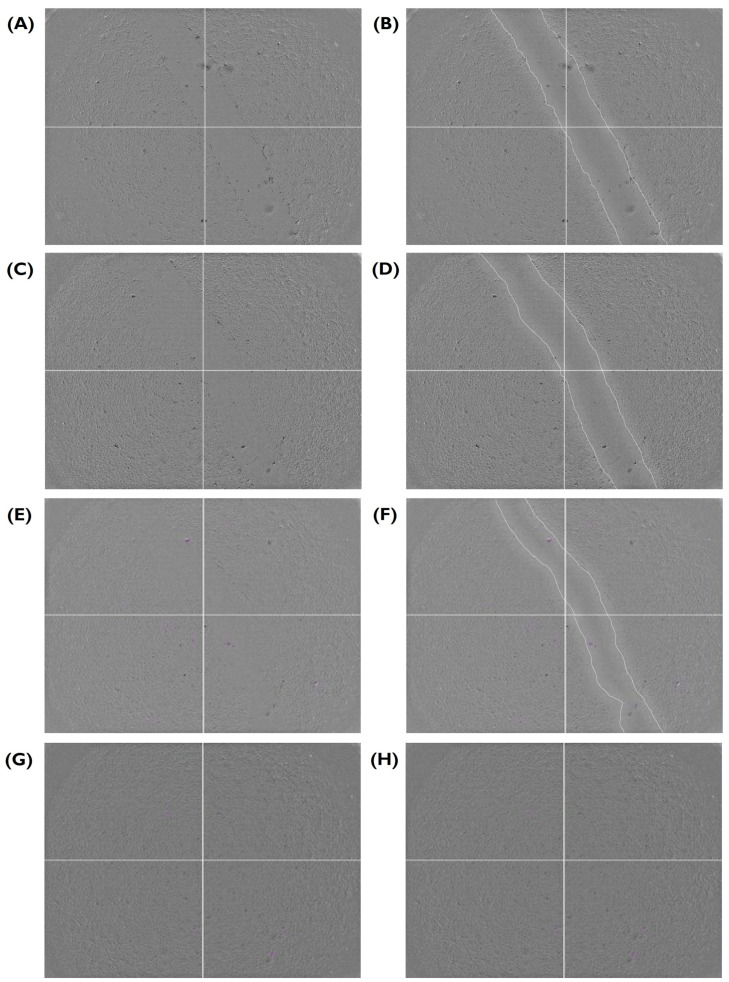
Closure of the gap area in a HUVEC monolayer over time, provided as a visual aid for Figure 5 and Figure 6. Here, fibrin in serum-free media at 2.5% *w*/*v* sample is shown (**A**) immediately after the wound was created and at (**C**) 1.5, (**E**) 6, and (**G**) 22 h. (**B**,**D**,**F**,**H**) A replicate of each image highlighting the edge between the cell monolayer and the wound is shown next to each original image. HUVECs were cultured with supplemented EGM-2 and seeded at a density of 1.5 × $10^{4}$ cells/well (96-well plate).

**Figure 5:**
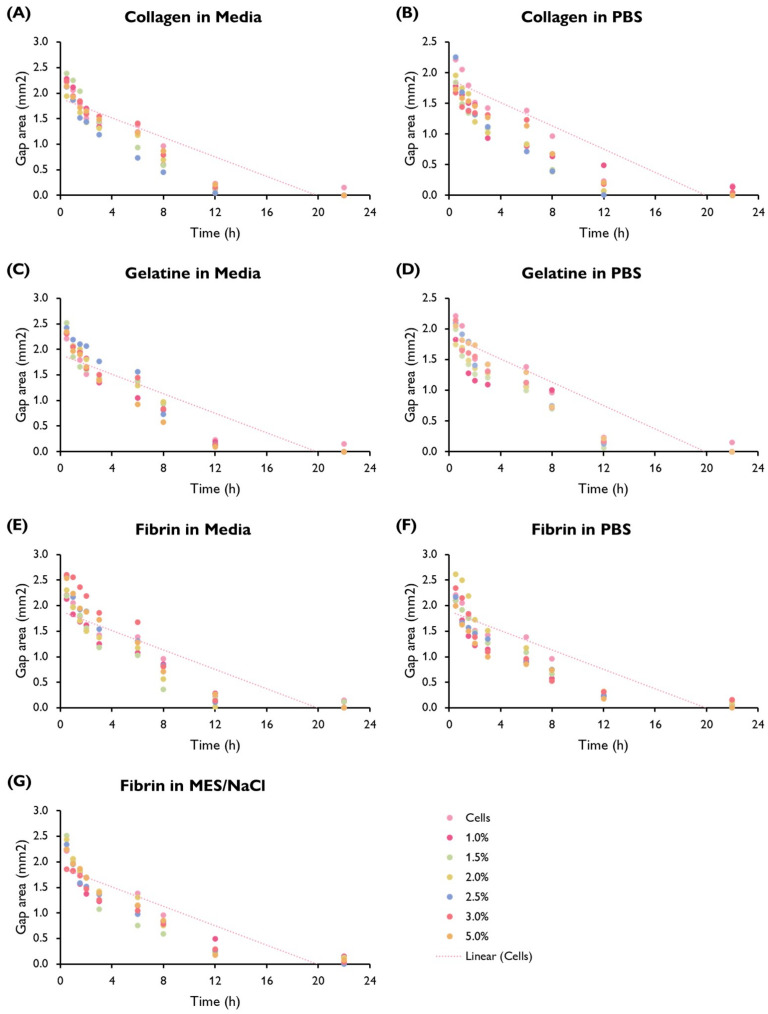
Gap area (mm${}^{2}$) in a HUVEC monolayer over time (h) by hydrogel composition showing the trend line fitted to the cells-only control (replicated in each graph). HUVECs were cultured with supplemented EGM-2 and seeded at a density of 1.5 × $10^{4}$ cells/well (96-well plate). (ANOVA/Tukey HSD, *p* < 0.05; *n* = 3).

**Figure 6:**
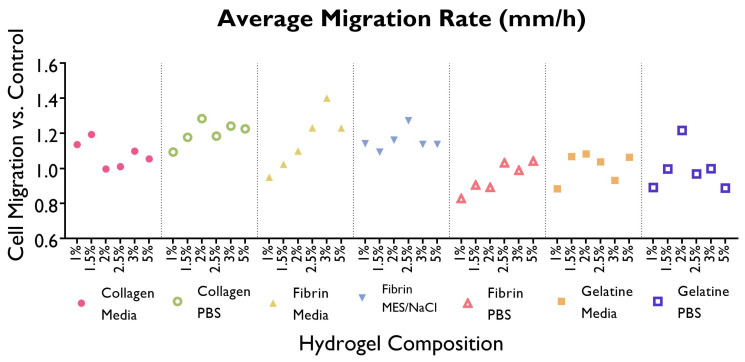
Average migration rate (μm/h) required to fill the wound in the HUVEC monolayer created using a pipette time is shown as a line graph, by gel composition. Cells were cultured with supplemented EGM-2 and seeded at a density of 1.5 × $10^{4}$ cells/well (96-well plate). (ANOVA/Tukey HSD, *p* < 0.05; *n* = 3).

**Figure 7:**
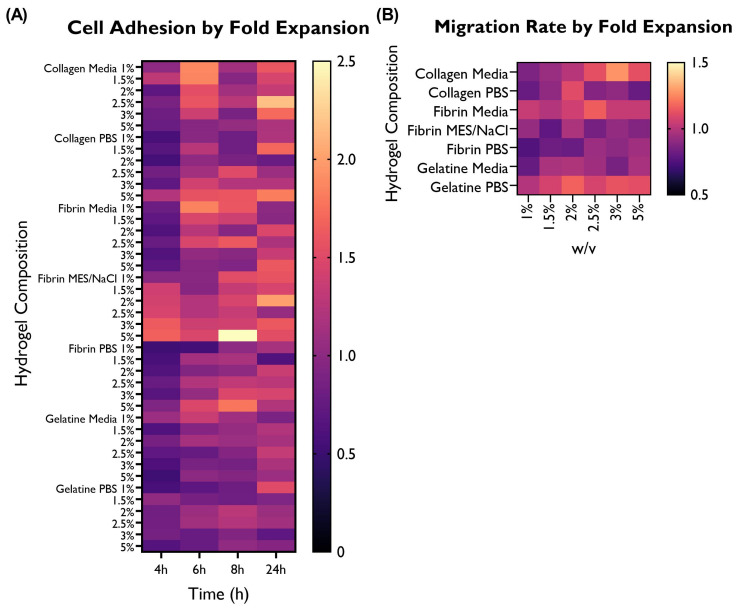
Overview of the performance of the different hydrogel compositions in comparison with the cells-only control, as fold expansion, in terms of (**A**) cell adhesion and (**B**) migration rate. HUVECs were cultured with supplemented EGM-2 and seeded at a density of 2 × $10^{4}$ or 1.5 × $10^{4}$ cells/well (96-well plate), respectively.

Following a scratch wound on the HUVEC monolayer, the gap area was measured immediately after the creation of the gap and at different time points (Figure 4 and Figure 5). From here, the slope generated by these data points was obtained and used to calculate the average migration rate and the estimated time required to fill the gap (Figure 6). A summary based on these data was drafted to facilitate a comparison of migration rates between gels (Figure 7).

Cells displayed a faster migration rate in the hydrogels containing serum-free media as a solvent rather than PBS or MES/NaCl. Interestingly, this trend is not mirrored by gelatine hydrogels, where a faster migration rate takes place in PBS hydrogels. No significant differences can be observed within the same hydrogel group (i.e., a specific polymer–solvent combination) when comparing different % *w*/*v* concentrations.

### 3.4. Selection of Hydrogel Compositions Based on Cell Adhesion and Migration Rate

Based on the results obtained from the cell adhesion and wound-healing assays (Figure 7), the gelatine was discarded for future experimental work. Furthermore, considering the promising data produced by fibrin and collagen hydrogels, a combination of these two at a 1:1 ratio was created and included in the following experimental phase.

### 3.5. Cell Viability

**Figure 8:**
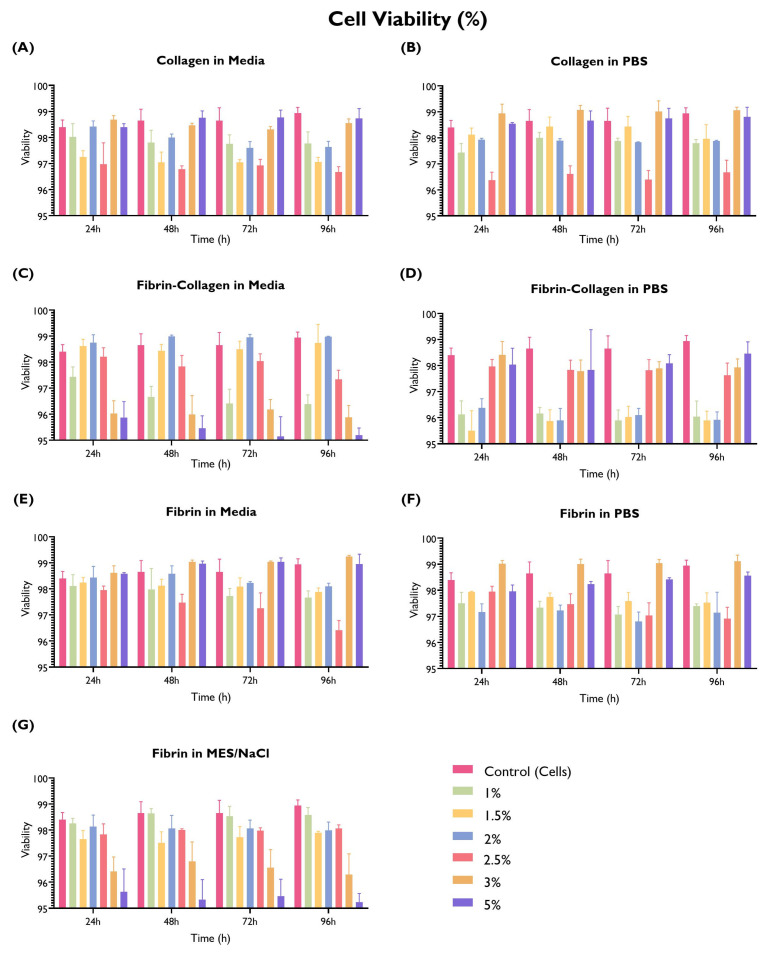
Cell viability (%) by hydrogel composition (fibrin, collagen, or fibrin and collagen at 1:1 ratio in PBS, serum-free media, or MES/NaCl (fibrin only) at a concentration of 1, 1.5, 2, 2.5, 3, or 5% *w*/*v*. HUVECs (seeding density: 2 × $10^{4}$ cells/well) were cultured with supplemented EGM-2 in 96-well plates on top of the hydrogel. Readings were taken at 24 h, 48 h, 72 h, and 96 h. A cells-only control (repeated in every graph for comparison purposes) was used. (ANOVA/Tukey HSD, *p* < 0.05; *n* = 3).

Fibrin and collagen gels, including a collagen–fibrin mix (1:1 ratio), were tested to evaluate cell viability at different time points (Figure 8).

Nearly an equal proportion of the tested gels delivered similar (45.2%) or lower (47.6%) cell viability in comparison with the cells-only control. Three gels were associated with a statistically significant higher cell viability than the control in at least one time point, with the remaining time points being equivalent to the control: collagen in PBS 3% *w*/*v*, fibrin in serum-free media 3% *w*/*v*, and fibrin in PBS 3% *w*/*v*.

Collagen shows similar cell viability in both PBS and serum-free media, except for the 2.5% *w*/*v* concentration, whereas fibrin in serum-free media exhibits higher cell viability or equivalent to the control (especially 3% and 5% *w*/*v*); when using MES/NaCl buffer, these concentrations show the lowest viability. Interestingly, the fibrin–collagen gel at a 1:1 ratio was associated with a lower cell viability, particularly when mixed with PBS at lower *w*/*v* concentrations. On the other hand, when mixed with serum-free media, lower cell viability is associated with higher *w*/*v* concentrations. It is possible that this difference can be associated with hydrogel consistency.

### 3.6. Hydrogel Consistency

**Figure 9:**
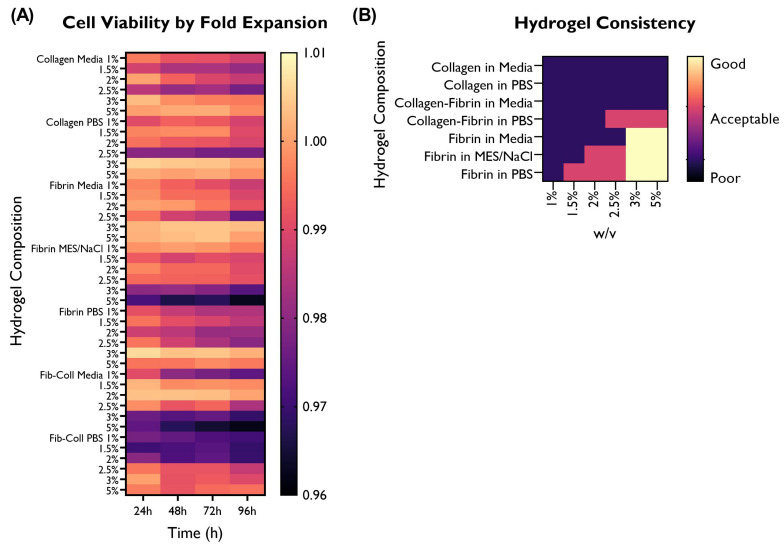
Overview of the performance of the different hydrogel compositions in comparison with the cells-only control, as fold expansion, in terms of (**A**) cell viability. (**B**) Hydrogel consistency was also visually evaluated, and rated as poor, acceptable, or good based on the desired characteristics for a 3D construct able to retain its architecture. HUVECs were cultured with supplemented EGM-2 and seeded at a density of 2 × $10^{4}$ cells/well (96-well plate).

The hydrogel consistency was visually inspected and categorised (Figure 9B). Hydrogels were either transparent (PBS and MES/NaCl) or pink (serum-free media). Furthermore, as the % *w*/*v* increased, hydrogels became more opaque. This was particularly noticeable in the ones using MES/NaCl buffer as a solvent.

In terms of hydrogel consistency, higher % *w*/*v* concentrations delivered more viscous gels and displayed better shape retention after 1 h. Furthermore, collagen hydrogels were less viscous than fibrin or fibrin–collagen ones at the same % *w*/*v* concentration.

Interestingly, none of the collagen hydrogels displayed acceptable or good shape retention. The only hydrogel compositions containing collagen that had an acceptable consistency and shape retention were collagen–fibrin in PBS at 2.5, 3, or 5% *w*/*v*.

Five fibrin hydrogels (in MES/NaCl: 2, 2.5%; in PBS: 1.5, 2, 2.5%) also displayed acceptable shape retention, whereas only high concentration fibrin hydrogels (3 and 5% *w*/*v*) had a good consistency. Relevantly, all solvents used for fibrin hydrogels displayed good shape retention, thus suggesting that both the % *w*/*v* and the material (fibrin) play a greater role in hydrogel consistency than the solvent.

### 3.7. Further Selection of Hydrogels Based on Cell Viability and Hydrogel Shape Retention

The optimal hydrogel composition was further refined based on cell viability and hydrogel consistency and shape retention (Figure 9).

Given that around half of the hydrogel compositions were associated with a similar or better cell viability than the control, hydrogel consistency was used to further refine the optimal hydrogel composition for the development of a scaffold for microvascular network formation. The collagen and fibrin–collagen hydrogels were discarded due to their poor shape retention.

From the remaining hydrogel compositions, the 3 and 5% *w*/*v* were selected as they exhibited a good hydrogel consistency and shape retention after removing the scaffold from the casting mould. From these six candidates (fibrin in media, fibrin in MES/NaCl, and fibrin in PBS), the two most promising hydrogel compositions were also selected considering cell adhesion and migration rate (Figure 7 and Figure 9). Based on the performance of the different hydrogel compositions, fibrin in serum-free media at 3 and 5% *w*/*v* were selected for further study.

### 3.8. Tube Formation

**Figure 10:**
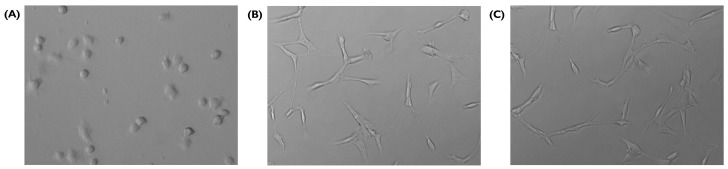
Tube formation on fibrin in serum-free media at 3% *w*/*v*, (**A**) 0.5 h, (**B**) 8 h, and (**C**) 10 h after seeding (20×). HUVECs were cultured with supplemented EGM-2 and seeded at a density of 2 × $10^{4}$ cells/well (96-well plate).

A tube formation assay was used to validate capillary-like network formation on fibrin in serum-free media (3% and 5% *w*/*v*) hydrogels. HUVECs developed networks of tube-like structures when seeded on these hydrogels (Figure 10). The formation of these structures is essential for the development of microvascular in vitro models. Networks were formed from 6 h and degraded by 24 h.

Once the networks were formed, characterisation including the number, length, and diameter of segments was carried out (Table 1). To gain further understanding of the overarching architecture created with the tube-like structures, segments were classified as linear, primary, or branches, and the arrangements between different types of segments (e.g., branch to primary segment ratio, the angle formed between primary segments and branches) were also characterised.

Tube-like structures arranged in a network were formed in both fibrin in serum-free media hydrogels, at both 3% and 5% *w*/*v*. Moreover, the networks formed on both hydrogels were similar in terms of the number, length, and diameter of segments as well as the network architecture. The average diameter of segments was similar between the 3% (8.6446 ± 3.0792) and 5% (7.8599 ± 2.3794) *w*/*v* concentrations.

The 3% and 5% hydrogel had a similar number of total segments: 10.3 ± 3.21 (3% *w*/*v*) vs. 9.6 ± 3.51 (5% *w*/*v*). In the 3% *w*/*v*, the average number of primary segments was slightly greater (2.66 vs. 2.33) whereas the branch number average was smaller (1.15 vs. 2). The average number of linear segments was exactly the same. Regarding the branch to primary segment ratios, 26.6% of the PS split into two branches whereas the remaining ones continued as a single segment, albeit following a change in the angle.

## 4. Discussion

The research outlined here presents the development and optimisation of the composition of a hydrogel microvasculature scaffold. A range of hydrogels was created from different combinations of materials (collagen, fibrin, gelatine), solvents (PBS, serum-free EBM-2 media, MES/NaCl buffer) and concentrations (1–5% *w*/*v*)). The optimisation process of the hydrogel composition comprises a multipronged evaluation combining cell adhesion, migration rate, cell viability, hydrogel consistency and shape retention, and tube formation. A large number of candidates were screened and systematically narrowed down based on the results obtained in the different experimental phases, therefore eliminating those hydrogel compositions that were suboptimal for the development of the construct.

The results obtained in the cell adhesion and migration rate phases clearly suggested that gelatine hydrogels using both serum-free media and PBS as solvents are not well suited for the development of this construct. Low cell adhesion across all weight/volume concentrations and the lack of a significantly higher associated migration rate enabled the decision to discard gelatine hydrogels from further experimental work.

Furthermore, hydrogels containing collagen also delivered substandard results in terms of cell viability and, particularly, shape retention and hydrogel consistency. Of all the hydrogel compositions containing collagen, 87.5% of them were associated with significantly lower cell viability values. Moreover, 75% (18 compositions) of the collagen-containing hydrogels exhibited such low values in at least three of the four time points. Furthermore, none of the collagen or collagen–fibrin hydrogels exhibited good shape retention or hydrogel consistency. In contrast, six of the fibrin hydrogels had good shape retention (3 and 5% *w*/*v* fibrin in all solvents). The experimental results suggest that the hydrogels containing collagen evaluated in this research are not optimal for the development of this construct, so these compositions were not explored further.

Following this multipronged optimisation strategy, we narrowed down the two most promising hydrogel compositions from the initial 42 candidates for the final experimental phase. Shape retention is a crucial characteristic in the development of the construct, as it will allow it to be removed from the mould to be used for further research as a free-standing platform. Therefore, only the hydrogel compositions that exhibited acceptable or good shape retention and hydrogel consistency could be considered promising candidates for the development of the construct. From the hydrogel compositions exhibiting good or acceptable shape retention and hydrogel consistency, the selection was made based on the results obtained in the cell viability, migration rate, and cell adhesion experimental phases.

Fibrin hydrogels in serum-free media at 3 and 5 % *w*/*v* were the most promising candidates, so their tube formation capability was evaluated. Both hydrogels enabled the formation of capillary-like networks, which is central to the development of a microvasculature construct. The results obtained in this research are further validated by similar findings in the literature. For instance, Teichert-Kuliszewska et al. [42] report successful HUVEC tube formation when seeded on fibrin gels, whereas Narayan et al. [43] confirm that all capillary-like vessel sprouting occurs by 24 h, after which no cell spreading takes place.

Relevantly, similar concentrations of fibrin (2.5 and 5) have been found to produce capillary-like networks with the greatest number of junctions and segments when compared with higher or lower concentrations [44]. However, as the networks produced in both hydrogels are similar to each other both in terms of the number of segments and microarchitecture, no clear preference for either hydrogel composition can be declared. Further refinement of the % *w*/*v* ought to consider long-term applications for these 3D microvasculature scaffolds and would benefit from including material characterisation.

As demonstrated in this work and supported by similar studies found in the literature, including Anderson et al. [45] and Hasenberg et al. [44], fibrin is a suitable scaffold material for the formation of capillary-like networks. Relevantly, fibrin supports angiogenesis in 2D and 3D environments, and in both single-cell and co-culture models, it is a promising option as the engineered construct’s ECM to promote desired cell functions.

In the body, fibrin plays an important role as a provisional cellular matrix during wound healing and tissue remodelling [9] and is also involved in cell adhesion and migration, blood clotting, and tissue morphogenesis [46]. Furthermore, its porosity, mechanical properties, and degradation rate can be controlled [46], permitting construct customisation [9]. This makes fibrin, a biodegradable matrix, particularly well-suited for tissue engineering applications due to its potential to allow for tissue regeneration.

The optimisation process of the hydrogel composition led to gelatine and collagen hydrogels being discarded from further experimental work. The results produced in this research supporting this strategy are further validated by the literature. For instance, collagen hydrogels exhibit poor mechanical properties [20,29] which can be improved, as well as its structural integrity, by combining it with another biomaterial [47]. On the other hand, gelatine hydrogels of concentrations lower than 10% *w*/*v* result in insufficient shape fidelity [47]. These findings further validate that both collagen and gelatine are suboptimal for the development of a free-standing 3D construct, which demands a biomaterial with good shape retention to enable its use in further applications, including as a testing platform for traumatic injury research.

The wound-healing assay is a standard in vitro technique used to evaluate collective cell migration [40]. This ‘sheet’ migration is exhibited by endothelial cell monolayers and was corroborated in the present experimental work. All the hydrogels tested, as well as the cells-only control, closed the gap created by the pipette tip essentially ‘healing’ the wound. Interestingly, cells displayed a faster migration rate in hydrogels using serum-free media as a solvent in comparison with those using PBS or MES/NaCl. This highlights the impact of the selection of a solvent which, as stated by Rosińska et al. [48], has a clear visible influence on the rigidity of the material obtained, impacting cell migration.

Other studies implementing similar methodologies for wound closure assays produce similar results regarding cell sheet migration. For instance, Anderson et al. [45] report that HUVECs seeded on fibrin gels closed the gap almost entirely by the end of the monitoring period (18 h). This is similar to the results obtained in this research for fibrin, collagen, and gelatine hydrogels, where the gap was closed after 20 h in the majority of compositions and after 22 h in all hydrogel variations. The cell migration rate is not only impacted by the hydrogel composition; environmental factors such as the suminsitration of high glucose [49] can accelerate cell migration.

The cell migration rate was obtained using the slope of the generated linear trendline, as suggested by the established protocols in the literature [40]. However, the pattern exhibited by all hydrogel compositions and the cells-only control suggests that there is a higher reduction in the gap area in the first hours, which then decelerates as time progresses. It would therefore be interesting to explore other types of trendlines to identify potential alternatives that fit the data better. This could provide a more accurate estimation of the area recovered by the cells at a given time point, considering an accelerating/decelerating migration rate across time.

Given that the wound was manually induced, it can be difficult to generate reproducible wounds. For instance, the angle at which the pipette tip is held plays a key role as there needs to be consistent pressure applied to create a consistent gap width. In order to increase reproducibility, an insert could be placed in the wells before cell seeding, ensuring that no cells can migrate into the space that will later on become the wound when removing the insert; although this could minimise the variation between wound size across samples, the removal of the insert could provoke a tear in the cell monolayer, leaving uneven gap edges.

Regarding cell viability, there seems to be no trend associated with the selection of PBS, MES/NaCl, or serum-free media; most differences in cell viability can be attributed to the choice of fibrin, collagen, or fibrin–collagen. Interestingly, the fibrin–collagen (1:1) hydrogels, particularly when mixed with PBS at lower % *w*/*v* concentrations, yielded lower cell viability. Further research is required, particularly exploring different fibrin–collagen ratios.

It is worth mentioning that the control yielded very high cell viability across all time points, potentially making it more challenging to identify hydrogels with a good performance. Nevertheless, the three materials tested at different *w*/*v* concentrations delivered good cell viability (>95%). This is expected, as these materials have been previously used for cell culture with positive outcomes and have been deemed biocompatible.

When evaluating hydrogel consistency and scaffold shape retention, the fibrin hydrogels outperformed both collagen and fibrin–collagen scaffolds, which had poor performance. Moreover, higher % *w*/*v* hydrogels produced more viscous gels and displayed better shape retention. Lower concentrations of fibrin hydrogels produced scaffolds with an acceptable consistency, whereas the 3 and 5% *w*/*v* hydrogels exhibited a good consistency.

Although more viscous gels are associated with better shape retention once demoulded, this could also translate into higher resistance for cell migration. On the other hand, hydrogels that are not viscous enough failed to retain their shape after removing them from the wells they were cast in. This limits their application to research that can be conducted on hydrogels contained in their casting moulds and would make it very challenging to produce a stand-alone 3D construct.

This research aims to develop and optimise 3D scaffolds for microvascular networks. However, a number of the experimental assays conducted as part of the optimisation of the hydrogel composition are carried out in 2D because some of the characteristics that are explored in this research can be best quantified in this way using standard in vitro procedures. For instance, the wound-healing assay is a standard technique for probing collective cell migration widely used in the tissue engineering field [49,50]. Nevertheless, the optimisation of the hydrogel composition carried out throughout this research leads to the successful formation of capillary-like networks in 3D environments. As addressed by Hasenberg et al. [44], the use of fibrin as the material for hydrogel fabrication does not limit the supply of oxygen and nutrients to the cells contained within, therefore allowing for 3D network formation throughout the gel.

HUVECs have been widely used for in vitro work as they are easily available and have demonstrated the ability to form new vasculature. Although they are considered the gold standard in endothelial cell line research, macrovascular cells differ from microvascular endothelial cells (MVECs) in a number of ways, including the prostaglandin secretory profile, types and amounts of cell adhesion molecules, cytoskeletal terms, and secreted proteins [46,51,52,53]. In particular, MVECs express specific proteins associated with tight junctions that are required to regulate capillary permeability [54]. As such, it would be interesting to utilise MVECs such as human dermal microvascular endothelial cells (HDMEC-1) for the development of a microvasculature construct that is more physiologically relevant.

There are a number of experimental methods used to carry out traumatic injury research, the majority of which employ in vivo models [55]. Nevertheless, modelling trauma in animal models entails major ethical concerns due to, among others, the mental stress or pain that they undergo. Moreover, although some in vivo models, like pigs, are chosen based on biomechanical and scale similarity, physiologic and pathophysiologic differences between animal models and humans remain [8]. These differences can make translating trauma research significantly more complex. Taking this into account, there is a need for physiologically relevant in vitro models that can be used for trauma research to provide not only a fundamental understanding of the mechanism of injury but also serve as platforms to test potential therapeutics for tissue repair and regeneration.

The research presented here addresses this gap by developing and optimising a hydrogel scaffold tailored for endothelial cells and, in particular, microvascular network formation. This construct not only enables the formation of tube-like structures arranged in a microvascular network, but the optimisation of its design also considers the characteristics required for using this construct as a platform for in vitro trauma research.

## 5. Conclusions

There is a need for in vitro models for microvasculature trauma research that allow us to gain a fundamental understanding of traumatic injuries and, at a later stage, test therapeutics to enhance tissue repair and regeneration. The selection of polymer type, ratio, and solvent impacts the properties of the resulting hydrogel. By optimising these parameters, this research aims to identify the hydrogel composition best suited for the development of microvasculature scaffolds. Constructs using a range of collagen, fibrin, or gelatine hydrogels and HUVECs were created and evaluated to identify the most promising composition. These hydrogels were compared based on their performance in terms of cell viability, migration rate, cell viability, hydrogel consistency, and the capacity to form tube-like structures. Based on the results produced in this research, the most promising candidates are fibrin in serum-free media at 3 and 5% *w*/*v*.

Further work on the optimisation of the construct is required in terms of material characterisation including, for instance, degradation. Moreover, it would be beneficial to incorporate additional cell lines into the construct in an effort to mimic the physiologic architecture of the natural tissue. Finally, the next step for this microvasculature hydrogel scaffold would be its use for traumatic injury in vitro research and validation.

## Figures and Tables

**Table 1:** Measurements obtained from tube formation assays, as average ± standard deviation.

Overall			
Hydrogel Composition	Segments (n)	Length (μm)	Diameter (μm)
Fibrin in Media 3%	14	82.5141 ± 31.8544	8.1661 ± 3.6087
	9	85.2777 ± 47.0683	6.0084 ± 4.0298
	8	106.9875 ± 79.0209	10.1942 ± 2.8000
Fibrin in Media 5%	13	71.8307 ± 33.8098	7.2011 ± 1.5661
	10	83.1000 ± 44.9076	7.1529 ± 4.9610
	6	254.2000 ± 61.9586	3.4445 ± 3.6139
Linear Segments			
Hydrogel Composition	Segments (n)	Length (μm)	Diameter (μm)
Fibrin in Media 3%	7	85.4857 ± 40.5427	6.84731 ± 2.1636
	2	114.7501 ± 78.4181	8.7435 ± 0.7881
	4	86.9250 ± 65.0272	10.5004 ± 2.1834
Fibrin in Media 5%	7	77.4714 ± 41.8278	7.5757 ± 1.9907
	2	114.7514 ± 78.4182	8.5380 ± 0.7778
	4	265.9250 ± 74.5172	7.0525 ± 0.5530
Primary Segments (PS)			
Hydrogel Composition	Segments (n)	Length (μm)	Diameter (μm)
Fibrin in Media 3%	3	95.0706 ± 11.9092	8.3272 ± 3.9164
	3	101.9666 ± 26.6421	8.9152 ± 1.57185
	2	150.1500 ± 156.0584	10.4133 ± 3.2766
Fibrin in Media 5%	3	79.8000 ± 18.2978	7.1394 ± 0.5376
	3	79.4333 ± 52.0003	10.8779 ± 5.9262
	1	209.7000	6.4110
Branches			
Hydrogel Composition	Segments (n)	Length (μm)	Diameter (μm)
Fibrin in Media 3%	4	9.3628 ± 5.2903	10.3534 ± 5.1363
	4	58.0250 ± 39.2037	6.9669 ± 2.6318
	2	103.9500 ± 36.2745	9.3628 ± 5.2903
Fibrin in Media 5%	3	50.7000 ± 19.9682	6.3886 ± 1.0253
	5	72.6400 ± 31.6308	8.6557 ± 1.8876
	1	251.8000	6.7137
PS and Branches Relation			
Hydrogel Composition	Angle (°)		Branch/PS Ratio
Fibrin Media 3%	49.9135 ± 18.3751		1.3333
	133.9715 ± 33.3319		1.3333
	27.8583 ± 34.6421		1
Fibrin Media 5%	23.5643 ± 26.3969		1
	72.3778 ± 12.8855		1.6667
	26.2000 ± 15.1868		1

## Data Availability

The data presented in this research are available upon request from the corresponding author.

## Abbreviations

The following abbreviations are used in this manuscript:

ANOVA	Analysis of Variance
ECM	Extracellular Matrix
EGM-2	Endothelial Cell Growth Medium-2
HDMEC-1	Human Dermal Microvascular Endothelial Cells
HUVECs	Human Umbilical Vein Endothelial Cell
HSD	Honest Significant Difference
MVECs	Microvascular Endothelial Cells
PBS	Phosphate-Buffered Saline
PS	Primary Segment
*w*/*v*	weight/volume

## References

[1] Sandsmark D.K., Bashir A., Wellington C.L., Diaz-Arrastia R. (2019). Cerebral Microvascular Injury: A Potentially Treatable Endophenotype of Traumatic Brain Injury-Induced Neurodegeneration. Neuron.

[2] Holtslag H.R., Van Beeck E.F., Lichtveld R.A., Leenen L.P., Lindeman E., Van Der Werken C. (2008). Individual and population burdens of major trauma in the Netherlands. Bull. World Health Organ..

[3] Diseases G., Colalborators I. (2020). Global burden of 369 diseases and injuries in 204 countries and territories, 1990–2019: A systematic analysis for the Global Burden of Disease Study 2019. Lancet.

[4] Haagsma J.A., Graetz N., Bolliger I., Naghavi M., Higashi H., Mullany E.C., Abera S.F., Abraham J.P., Adofo K., Alsharif U. (2016). The global burden of injury: Incidence, mortality, disability-adjusted life years and time trends from the global burden of disease study 2013. Inj. Prev..

[5] Javali R.H., Krishnamoorthy, Patil A., Srinivasarangan M., Suraj, Sriharsha (2019). Comparison of injury severity score, new injury severity score, revised trauma score and trauma and injury severity score for mortality prediction in elderly trauma patients. Indian J. Crit. Care Med..

[6] Kehoe A., Smith J.E., Edwards A., Yates D., Lecky F. (2015). The changing face of major trauma in the UK. Emerg. Med. J..

[7] Bruijns S.R., Guly H.R., Bouamra O., Lecky F., Lee W.A. (2013). The value of traditional vital signs, shock index, and age-based markers in predicting trauma mortality. J. Trauma Acute Care Surg..

[8] Weber B., Lackner I., Haffner-Luntzer M., Palmer A., Pressmar J., Scharffetter-Kochanek K., Knöll B., Schrezenemeier H., Relja B., Kalbitz M. (2019). Modeling trauma in rats: Similarities to humans and potential pitfalls to consider. J. Transl. Med..

[9] Ceccarelli J., Putnam A.J. (2014). Sculpting the blank slate: How fibrin’s support of vascularization can inspire biomaterial design. Acta Biomater..

[10] Guilak F., Butler D.L., Goldstein S.A., Baaijens F.P. (2014). Biomechanics and mechanobiology in functional tissue engineering. J. Biomech..

[11] Valarmathi M.T., Fuseler J.W., Potts J.D., Davis J.M., Price R.L. (2018). Functional Tissue Engineering: A Prevascularized Cardiac Muscle Construct for Validating Human Mesenchymal Stem Cells Engraftment Potential in Vitro. Tissue Eng. Part A.

[12] Zimmermann W.H., Fink C., Kralisch D., Remmers U., Weil J., Eschenhagen T. (2000). Three-dimensional engineered heart tissue from neonatal rat cardiac myocytes. Biotechnol. Bioeng..

[13] Gholobova D., Decroix L., Van Muylder V., Desender L., Gerard M., Carpentier G., Vandenburgh H., Thorrez L. (2015). Endothelial network formation within human tissue-engineered skeletal muscle. Tissue Eng. Part A.

[14] Moya M.L., Hsu Y.H., Lee A.P., Christopher C.W., George S.C. (2013). In vitro perfused human capillary networks. Tissue Eng. Part C Methods.

[15] Traore M.A., George S.C. (2017). Tissue Engineering the Vascular Tree. Tissue Eng. Part B.

[16] Bogorad M.I., DeStefano J., Wong A.D., Searson P.C. (2017). Tissue-engineered 3D microvessel and capillary network models for the study of vascular phenomena. Microcirculation.

[17] Liu Y.X., Xu B.W., Niu X.D., Chen Y.J., Fu X.Q., Wang X.Q., Yin C.L., Chou J.Y., Li J.K., Wu J.Y. (2022). Inhibition of Src/STAT3 signaling-mediated angiogenesis is involved in the anti-melanoma effects of dioscin. Pharmacol. Res..

[18] Li X., Wang C., Zhang H., Li Y., Hou D., Liu D., Xu R., Cheng J., Liu L.K., Fu Y. (2023). circFNDC3B Accelerates Vasculature Formation and Metastasis in Oral Squamous Cell Carcinoma. Cancer Res..

[19] Nelson D.W., Gilbert R.J. (2021). Extracellular Matrix-Mimetic Hydrogels for Treating Neural Tissue Injury: A Focus on Fibrin, Hyaluronic Acid, and Elastin-Like Polypeptide Hydrogels. Adv. Healthc. Mater..

[20] Ho T.C., Chang C.C., Chan H.P., Chung T.W., Shu C.W., Chuang K.P., Duh T.H., Yang M.H., Tyan Y.C. (2022). Hydrogels: Properties and Applications in Biomedicine. Molecules.

[21] Peppas N.A., Hilt J.Z., Khademhosseini A., Langer R. (2006). Hydrogels in biology and medicine: From molecular principles to bionanotechnology. Adv. Mater..

[22] Rivest C., Morrison D.W., Ni B., Rubin J., Yadav V., Mahdavi A., Karp J.M., Khademhosseini A. (2007). Microscale hydrogels for medicine and biology: Synthesis characteristics and applications. J. Mech. Mater. Struct..

[23] Geckil H., Xu F., Zhang X., Moon S., Demirci U. (2010). Engineering hydrogels as extracellular matrix mimics. Nanomedicine.

[24] Eyrich D., Brandl F., Appel B., Wiese H., Maier G., Wenzel M., Staudenmaier R., Goepferich A., Blunk T. (2007). Long-term stable fibrin gels for cartilage engineering. Biomaterials.

[25] Jarrell D.K., Vanderslice E.J., Lennon M.L., Lyons A.C., VeDepo M.C., Jacot J.G. (2021). Increasing salinity of fibrinogen solvent generates stable fibrin hydrogels for cell delivery or tissue engineering. PLoS ONE.

[26] Ren Y., Zhang H., Wang Y., Du B., Yang J., Liu L., Zhang Q. (2021). Hyaluronic Acid Hydrogel with Adjustable Stiffness for Mesenchymal Stem Cell 3D Culture via Related Molecular Mechanisms to Maintain Stemness and Induce Cartilage Differentiation. ACS Appl. Bio Mater..

[27] Wang Q., Wang X., Feng Y. (2023). Chitosan Hydrogel as Tissue Engineering Scaffolds for Vascular Regeneration Applications. Gels.

[28] Gillette B.M., Jensen J.A., Tang B., Yang G.J., Bazargan-Lari A., Zhong M., Sia S.K. (2008). In situ collagen assembly for integrating microfabricated three-dimensional cell-seeded matrices. Nat. Mater..

[29] Kilmer C.E., Battistoni C.M., Cox A., Breur G.J., Panitch A., Liu J.C. (2020). Collagen Type I and II Blend Hydrogel with Autologous Mesenchymal Stem Cells as a Scaffold for Articular Cartilage Defect Repair. ACS Biomater. Sci. Eng..

[30] Moxon S.R., Corbett N.J., Fisher K., Potjewyd G., Domingos M., Hooper N.M. (2019). Blended alginate/collagen hydrogels promote neurogenesis and neuronal maturation. Mater. Sci. Eng. C.

[31] Smidsrød O., Skjåk-Braek G. (1990). Alginate as immobilization matrix for cells. Ind. Eng. Chem. Process Des. Dev..

[32] Zhang W., Jiang Y., Wang H., Li Q., Tang K. (2022). In situ forming hydrogel recombination with tissue adhesion and antibacterial property for tissue adhesive. J. Biomater. Appl..

[33] Cosola A., Chiappone A., Martinengo C., Grützmacher H., Sangermano M. (2019). Gelatin Type A from Porcine Skin Used as Co-Initiator in a Radical Photo-Initiating System. Polymers.

[34] Kaur S., Kaur I., Rawal P., Tripathi M., Vasudevan A. (2021). Non-matrigel scaffolds for organoid cultures. Cancer Lett..

[35] Habanjar O., Diab-Assaf M., Caldefie-Chezet F., Delort L. (2021). 3D cell culture systems: Tumor application, advantages, and disadvantages. Int. J. Mol. Sci..

[36] Kozlowski M.T., Crook C.J., Ku H.T. (2021). Towards organoid culture without Matrigel. Commun. Biol..

[37] Li X., Zhang Q., Yu S.M., Li Y. (2023). The Chemistry and Biology of Collagen Hybridization. J. Am. Chem. Soc..

[38] Lim X., Potter M., Cui Z., Dye J.F. (2018). Manufacture and characterisation of EmDerm—novel hierarchically structured bio-active scaffolds for tissue regeneration. J. Mater. Sci. Mater. Med..

[39] Sharma V., Patel N., Dye J.F., Hook L., Mason C., García-Gareta E. (2015). Albumin removal from human fibrinogen preparations for manufacturing human fibrin-based biomaterials. Biochim. Open.

[40] Jonkman J.E., Cathcart J.A., Xu F., Bartolini M.E., Amon J.E., Stevens K.M., Colarusso P. (2014). An introduction to the wound healing assay using live-cell microscopy. Cell Adhes. Migr..

[41] Schindelin J., Arganda-Carreras I., Frise E., Kaynig V., Longair M., Pietzsch T., Preibisch S., Rueden C., Saalfeld S., Schmid B. (2012). Fiji: An open-source platform for biological-image analysis. J. Nat. Methods.

[42] Teichert-Kuliszewska K., Maisonpierre P.C., Jones N., Campbell A.I., Master Z., Bendeck M.P., Alitalo K., Dumont D.J., Yancopoulos G.D., Stewart D.J. (2001). Biological action of angiopoietin-2 in a fibrin matrix model of angiogenesis is associated with activation of Tie2. Cardiovasc. Res..

[43] Narayan R., Agarwal T., Mishra D., Maiti T.K., Mohanty S. (2018). Goat tendon collagen-human fibrin hydrogel for comprehensive parametric evaluation of HUVEC microtissue-based angiogenesis. Colloids Surf. Biointerfaces.

[44] Hasenberg T., Mühleder S., Dotzler A., Bauer S., Labuda K., Holnthoner W., Redl H., Lauster R., Marx U. (2015). Emulating human microcapillaries in a multi-organ-chip platform. J. Biotechnol..

[45] Anderson S.M., Siegman S.N., Segura T. (2011). The effect of vascular endothelial growth factor (VEGF) presentation within fibrin matrices on endothelial cell branching. Biomaterials.

[46] Tiruvannamalai Annamalai R., Rioja A.Y., Putnam A.J., Stegemann J.P. (2016). Vascular Network Formation by Human Microvascular Endothelial Cells in Modular Fibrin Microtissues. ACS Biomater. Sci. Eng..

[47] Benwood C., Chrenek J., Kirsch R.L., Masri N.Z., Richards H., Teetzen K., Willerth S.M. (2021). Natural biomaterials and their use as bioinks for printing tissues. Bioengineering.

[48] Rosińska K., Bartniak M., Wierzbicka A., Sobczyk-Guzenda A., Bociaga D. (2023). Solvent types used for the preparation of hydrogels determine their mechanical properties and influence cell viability through gelatine and calcium ions release. J. Biomed. Mater. Res. Part B Appl. Biomater..

[49] Zhuang J., Gao P., Chen H., Fang Z., Zheng J., Zhu D., Hou J. (2022). Extracellular vesicles from human urine-derived stem cells merged in hyaluronic acid ameliorate erectile dysfunction in type 2 diabetic rats by glans administration. Andrology.

[50] Zuo L., Zhu S., Gu S., Xu X. (2023). Anti-scarring effects of conbercept on human Tenon’s fibroblasts: Comparisons with bevacizumab. BMC Ophthalmol..

[51] Patan S. (2000). Vasculogenesis and angiogenesis as mechanisms of vascular network formation, growth and remodeling. J. Neuro-Oncol..

[52] Karamysheva A.F. (2008). Mechanisms of angiogenesis. Nature.

[53] Kutcher M.E., Herman I.M. (2009). The pericyte: Cellular regulator of microvascular blood flow. Microvasc. Res..

[54] Bonnefoy A., Harsfalvi J., Pfliegler G., Fauvel-Lafève F., Legrand C. (2001). The subendothelium of the HMEC-1 cell line supports thrombus formation in the absence of von Willebrand factor and collagen types I, III and VI. Thromb. Haemost..

[55] de Souza J., Gottfried C. (2013). Muscle injury: Review of experimental models. J. Electromyogr. Kinesiol..

